# Recurrent Intussusception: Management Options

**Published:** 2011-03-10

**Authors:** Bilal Mirza

**Affiliations:** Department of Pediatric Surgery, The Children's Hospital and the Institute of Child Health Lahore, Pakistan

**Dear Sir**

Intussusception is referred to as recurrent when after being spontaneously reduced it is re-diagnosed clinically and on ultrasound, later confirmed at operation; or it is a post-operative intussusception in a patient where it was reduced at an open surgery or non-operatively either with hydrostatic or pneumatic method [1, 2, 3].

Most of the recurrent intussusceptions present within 6 months of initial episode and have been observed with almost all the treatment modalities. These are frequently reported after non-operative reduction (hydrostatic/pneumatic), whereas their incidence is negligible after operative management. This is especially true where the resection of the involved part of intestine is performed. The recurrence may occur once to as many as 8 times or more [1, 2, 3].

There is ongoing debate as to the management of recurrence. The recommended management is non-operative reduction, however, a significant number of recurrent intussusceptions do have pathological leads point (PLP); therefore operative management is required in these cases [1]. The purpose of this letter is to discuss various management options available for the treatment of recurrent intussusceptions based upon an experience of three cases of recurrent intussusception. 

**Case 1:**

 A 3-month-old male infant presented with abdominal pain, two episodes of bilious vomiting, irritability, and passage of currant jelly stool for two days. There was a preceding history of diarrhea. Clinically a sausage shaped mass was present in the lower abdomen. Ultrasound of the abdomen confirmed it as intussusception. At operation, no intussusception was found and appeared to be spontaneously reduced as indicated by hyperemia of a portion of distal ileum and collapse of the ileum distal to edematous segment.

On the third post-operative day the infant again developed similar features with toxic features. Patient underwent re-operation and resection of gangrenous ileum with primary end to end ileo-ileal anastomosis performed for ileo-ileal intussusception. This time, the Post-operative course remained uneventful. There was no recurrence during a follow up of 15 months.

**Case 2:**

 A 6-m-old male infant presented with abdominal pain, vomiting, and passage of currant jelly stool. The sausage shaped mass was palpable in the right upper quadrant. Ultrasound abdomen also revealed a positive pseudo-kidney sign. At operation ileo-colic intussusception found that was reduced manually. No PLP was detected. On 4th post operative day the clinical features of intestinal obstruction appeared. A diagnosis of recurrent intussusceptions was made and hydrostatic reduction with saline enema under ultrasound guidance was attempted. The intussusception was successfully reduced and the post-operative course remained uneventful. The patient had no further recurrence and doing well at 18 months of follow up.

**Case 3:**

 A 7-year-old boy presented with colicky abdominal pain, bilious vomiting, and bleeding per rectum for 3 days. There was no history of any preceding illness. Ultrasound done a day earlier suggested intussusception as suspected on clinical examination which revealed a sausage shaped mass in the right iliac and lumbar regions. The mass was spontaneously reduced within an hour of admission to our hospital. The symptoms again developed on the next day with the mass detectable clinically and demonstrated on ultrasound. The patient was planned for exploratory laparotomy with a strong suspicion of PLP. On operation table under general anesthesia the mass was not palpable. It was decided to proceed with laparotomy. Operative signs of spontaneous reduction of ileo-colic intussusception were found but no PLP demonstrated. Appendectomy was added with a view of probable PLP. The post operative course was uneventful. The patient remained asymptomatic at 3 months follow up.

The incidence of recurrent intussusceptions is 8-15% in most of the big series. The recurrence is higher with hydrostatic and pneumatic reduction (10-15%) and lower with operative reduction (1-3%). Post operative adhesions play a vital role in preventing recurrence. The recurrence is almost nil after resection of the involved portion of intestine which indicates that the involved part of intestine, even in absence of an obvious PLP, has a key role in development of recurrence [1].

Niramis et al have reported that about 70% of patients with recurrent intussusceptions were infants with a maximum incidence within 6 months of the initial episode. Frequently recurrent intussusceptions may have PLP as a cause of recurrence. Previously we have reported a case of recurrent colocolic intussusception in a 10 year old boy having inflammatory fibroid polyp as a PLP. In contrary to that, Niramis et al found maximum of 5 recurrences in two of their patients but neither of them had PLP when explored for it [1, 4].

The management of recurrent intussusception is somewhat tricky. One cannot exactly anticipate the future episodes of recurrences. Many authors are in favor of operative interventions in case of more than one episodes of recurrence in patients older than 2 years. As the recurrence is also reported after operative reduction therefore some authors recommend ileocolopexy for prevention of recurrence however others do not recommend it. Some authors suggested resection of involved portion of intestine in case of multiple recurrences [1, 5,6].

In summary, recurrent intussusceptions may pose difficulties as to the management. The selection of the treatment modality should be individualized as per case requirement.

## Footnotes

**Source of Support:** Nil

**Conflict of Interest:** None declared
